# Titin isoform size is not correlated with thin filament length in rat skeletal muscle

**DOI:** 10.3389/fphys.2014.00035

**Published:** 2014-02-03

**Authors:** Marion L. Greaser, Jonathan M. Pleitner

**Affiliations:** Muscle Biology Laboratory, Animal Sciences, University of Wisconsin-MadisonMadison, WI, USA

**Keywords:** titin, actin, phalloidin, tropomodulin, thin filament length

## Abstract

The mechanisms controlling thin filament length (TFL) in muscle remain controversial. It was recently reported that TFL was related to titin size, and that the latter might be involved in TFL determination. Titin plays several crucial roles in the sarcomere, but its function as it pertains to the thin filament has not been explored. We tested this relationship using several muscles from wild type rats and from a mutant rat model (Greaser et al., 2008) which results in increased titin size. Myofibrils were isolated from skeletal muscles [extensor digitorum longus (EDL), external oblique (EO), gastrocnemius (GAS), longissimus dorsi (LD), psoas major (PM), and tibialis anterior(TA)] using both adult wild type (WT) and homozygous mutant (HM) rats (*n* = 6 each). Phalloidin and antibodies against tropomodulin-4 (Tmod-4) and nebulin's N-terminus were used to determine TFL. The WT rats studied express skeletal muscle titin sizes ranging from 3.2 to 3.7 MDa, while the HM rats express a giant titin isoform sized at 3.8 MDa. No differences in phalloidin based TFL, nebulin distance, or Tmod distance were observed across genotypes. However, the HM rats demonstrated a significantly increased (*p* < 0.01) rest sarcomere length relative to the WT phenotype. It appears that the increased titin size, predominantly observed in HM rats' middle Ig domain, allows for increased extensibility. The data indicates that, although titin performs many sarcomeric functions, its correlation with TFL and structure could not be demonstrated in the rat.

## Introduction

**Figure 1:**
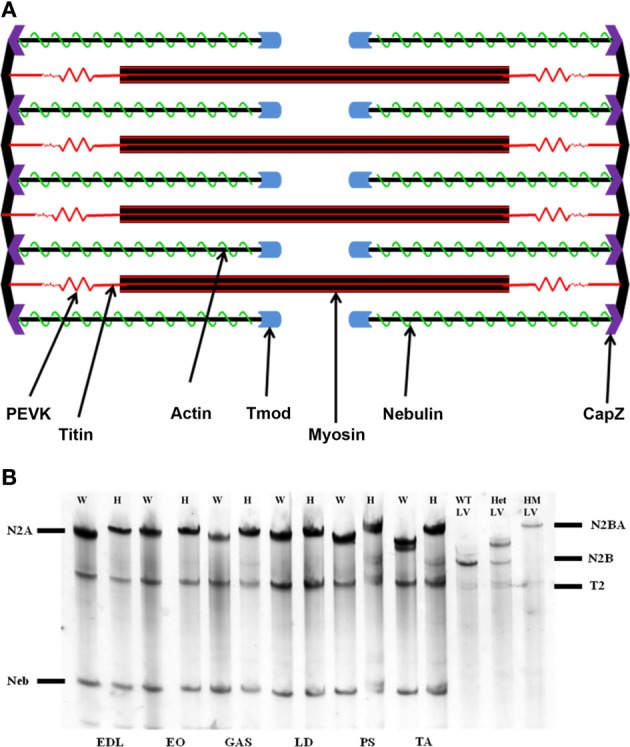
**(A)** Schematic representation of the sarcomere. The actin thin filament is capped at its barbed end by CapZ, and at its pointed end by tropomodulin (Tmod). Nebulin runs along the thin filament to act as a stabilizing protein. **(B)** Agarose SDS gel showing skeletal muscle titin isoform expression. EDL, extensor digitorum longus; EO, external oblique; GAS, gastrocnemius; LD, longissimus dorsi; PS, psoas major; TA, tibialis anterior; W, rat left ventricle; HM LV, homozygote rat left ventricle. The N2BA isoform is found only within cardiac tissue, while the N2A isoform comprises the totality of skeletal muscle titin. The mutant rat phenotype exhibits a giant titin isoform of 3.9 MDa.

One of the hallmarks of the striated muscle sarcomere is its precise periodicity, composed of a number of proteins held in tight alignment. The sarcomeric filament systems (thick, thin, titin, and nebulin) are optimized for specific functionalities in the muscle, and their relationship is key for contraction (Figure 1A). Because of the precise control necessary for contractile function, a multitude of protein-protein interactions must be maintained. The association between the sarcomere's thick and thin filaments has been established for decades (Huxley and Hanson, 1954; Huxley and Niedergerke, 1954), and the dynamic nature of the actin thin filament plays an essential role in this organization. In contrast to other F-actin environments, the actin of striated muscle primarily exhibits shrinkage and elongation from its pointed end, in the central region of the sarcomere (Littlefield et al., 2001; Mardahl-Dumesnil and Fowler, 2001). While the actin thin filament length (TFL) can vary across and within muscles of a species, its length within a sarcomere changes very little (Fowler, 1996).

In order for TFL to be specified, capping and ruler proteins must be present to ensure that this occurs. Both ends of the thin filament are capped to provide specified lengths and structural stabilization. CapZ binds the thin filament barbed end at the Z-disk, and tropomodulin (Tmod) caps actin at its pointed end (Fowler et al., 1993). At the barbed end, CapZ has been shown to be important in filament organization (Schafer et al., 1995), but not TFL determination. The pointed end of the thin filament requires Tmod and nebulin for length specification. Tmod is a ~40 kDa protein that originally was shown to bind tropomyosin (Fowler, 1987, 1990), but its interaction with the actin filament and nebulin is not fully understood (Gregorio et al., 1995; McElhinny et al., 2001). Indeed, it is Tmod's tropomyosin-binding ability that is believed to confer stability upon the thin filament and prevent disassembly. Further, it was shown that these protein-protein interactions are keys to the thin filament's pointed end dynamics.

Nebulin is a 500–900 kDa protein with actin-binding super-repeats that provide thin filament stability (McElhinny et al., 2003) and has long been believed to be the “molecular ruler” of the actin filament (Kruger et al., 1991; Labeit et al., 1991). Nebulin's N-terminus is capable of binding Tmod, which suggests that nebulin extends to the end of the thin filament (McElhinny et al., 2001). Upon discovery of nebulin's importance in actin nucleation and stabilization (Chen et al., 1993), the mechanisms of these actions have been studied extensively. Multiple models have been proposed in which nebulin is unable to reach the pointed end, intimating that this interaction may not exist (Littlefield and Fowler, 2008). However, both nebulin and Tmod are believed to be required for TFL specification.

Multiple manipulations to nebulin and Tmod expression levels have been made, providing new insights on their roles in TFL regulation. A nebulin knockout mouse has been produced, which results in early postnatal mortality. Subsequent studies indicate that, upon nebulin ablation, the sarcomeric organization is perturbed. Additionally, this phenotype demonstrates shorter thin filaments, and that nebulin is responsible for maintaining a minimal TFL via a stabilization mechanism (Bang et al., 2006; Witt et al., 2006; Gokhin et al., 2009; Pappas et al., 2010). Since Tmod's discovery as a capping protein (Fowler et al., 1993), the mechanism by which it performs this role has been ascertained. It can bind to both actin and tropomyosin to provide thin filament support (Mudry et al., 2003). Up-regulating Tmod expression leads to shortened thin filaments, lowering its levels causes elongated thin filaments (Sussman et al., 1998), and inactivating it via antibody microinjection extended TFL as well (Gregorio et al., 1995). Some combination of nebulin and Tmod control thus appears to be the primary causal agent in TFL determination, though the interplay between these proteins remains unclear.

The giant protein titin (Wang et al., 1979) performs a number of functions throughout the sarcomere (Lange et al., 2006; Linke, 2008; Tskhovrebova and Trinick, 2010), and spans from the sarcomere's Z-disk to its M-line. Despite being comprised of a single gene (Labeit and Kolmerer, 1995), titin possesses a wide array of splice variants, depending on the muscle and its physiological role (Guo et al., 2010). Generally speaking, skeletal muscle titin isoforms shrink in size as animals mature (Li and Greaser, unpublished data). Skeletal muscle contains the N2A titin isoform, but sizes of this isoform can vary from 3.3 to 3.7 MDa in the rabbit (Prado et al., 2005). A great deal of the alternative splicing events occur in the extensible PEVK and middle Ig regions of titin (Labeit and Kolmerer, 1995; Greaser et al., 2002). The splice variants are manifest primarily in titin's flexible region, suggesting that larger titin molecules would alter the sarcomere's filament system. Titin is the major protein responsible for passive tension maintenance, acting to prevent overstretch of the sarcomere, which is dependent upon the isoform size expressed (Neagoe et al., 2003). Our lab has serendipitously discovered a novel and spontaneous rat mutation which modifies titin splicing, causing a 3.9 MDa cardiac titin isoform to be expressed (Greaser et al., 2005, 2008), the largest titin molecule known to date. Therefore, we were curious if this significant increase in titin size would affect the sarcomeric structure, particularly the TFL.

Recently, a correlation between titin size and Tmod distance from the Z-disk was reported in the rabbit (Castillo et al., 2009), suggesting that titin may play a role in thin filament organization. Titin interacts directly with actin (Trombitas et al., 1997; Kulke et al., 2001; Nagy et al., 2004; Raynaud et al., 2004) and nebulin (Ma and Wang, 2002; Witt et al., 2006) at its I-band and Z-disk regions, respectively. Because of the splice variants that arise primarily out of the I-band domains, it was hypothesized that a rat model that does not exhibit a normal isoform transition would affect the thin filament. Our rat mutation serves as a good model to study titin's size effects, and this study was conducted to elucidate the effect, if any, an elongated titin protein has on TFL. Here, we use immunofluorescence and phase contrast microscopy to determine if titin size affects thin filaments lengths as estimated by phalloidin and Tmod staining.

## Materials and methods

### Animals and tissue collection

Two rat phenotypes were employed in this study: a wild type (WT) and a homozygous mutant (HM) phenotype with an abnormal titin splicing pattern. Both phenotypes were from a mixed Sprague-Dawley, Fisher 344, and Brown Norway crossbred strain. The mutant phenotype has been described previously (Greaser et al., 2005, 2008). Adult rats aged greater than 3 months were sacrificed and their muscles were collected. The skeletal muscles selected for this study were: extensor digitorum longus (EDL), external oblique (EO), gastrocnemius (GAS), longissimus dorsi (LD), psoas major (PM), and tibialis anterior (TA). Immediately following death, the skeletal muscles were excised and placed on ribbed cable ties. The muscle pieces were gently stretched and tied down with floss. At this point, the pair of muscles was separated according to the necessary treatment. For those muscles used in immunofluorescence, they were placed in a rigor buffer (RB) solution containing (in mM): 75 KCl, 5 K_2_HPO_4_, 2 EGTA, 2 MgCl_2_, 1 NaN_3_, 0.5 phenylmethylsulfonyl fluoride (PMSF), 0.5% Triton-X 100, pH 7.2, stirred gently and dialyzed overnight at 4°C. RB used in this study contained all of the above, excluding the PMSF and Triton-X 100. The myofibrils were homogenized and centrifuged through several cycles, as described by Wang and Greaser (1985), with several changes. Only Dounce homogenizers were used, and the myofibrils were stored at −20°C in 50% glycerol until use. The muscle pair used for resting sarcomere length (RSL) determination was placed unstretched on a cable tie and tied at both ends, and stored in relaxing buffer (in mM): 100 KCl, 20 Imidazole, 7 MgCl_2_, 2 EGTA, 4 ATP, 20 2,3-Butanedione-monoxime, 0.1 PMSF, and 0.2% Triton-X 100. The muscle strips were stirred gently overnight in relaxing buffer at 4°C. The samples were then put into the same buffer without PMSF and Triton-X 100, and glycerol was added to 50%. The muscle pieces were stored at −20°C for at least 24 h, then the tissues were removed and fiber bundles were teased apart with dissecting needles. The fibers were placed on a microscope slide and covered with a coverslip. Analysis with an inverted microscope directly followed.

### Agarose gel electrophoresis

The muscles listed above were also excised from adult rats, frozen in liquid nitrogen, and stored at −80°C. The frozen muscle tissue was dissolved and homogenized (with a Dounce homogenizer) in urea-thiourea-SDS buffer. Following electrophoresis, the gels were silver-stained according to the previously published procedure (Warren et al., 2003) to determine titin isoform expression.

### Myofibril immunofluorescence and microscopy

Three hundred microliter of glycerinated myofibril sample was mixed with 500 μl of RB containing 1 mg/ml bovine serum albumin (BSA). The mixture was centrifuged for 10 s, and the supernatant was aspirated. Seventy microliter of RB/BSA was added to the myofibrils, and the solution was placed on a coverslip, then let sit for 5 min. The coverslip was dipped in fresh RB, and then placed on a 5% goat serum in RB solution for 10 min. After this step, the myofibrils were put in an RB/BSA solution containing fluorescein isothiocyanate (FITC)-labeled phallodin (Sigma Aldrich, St. Louis, MO). Following a 15 min incubation at room temperature, the myofibrils were incubated with either nebulin or Tmod primary antibody. The nebulin antibody (NebN), which reacts against the first three N-terminal domains of the nebulin protein (McElhinny et al., 2001), was added to RB/BSA at a concentration of 1:1500 (rabbit 1357L, kindly provided by Dr. Carol Gregorio, University of Arizona, Tucson, AZ). NebN was incubated for 1 h at 4°C and rinsed with RB. A secondary antibody of anti-rabbit Texas Red (Sigma Aldrich, St. Louis, MO) at 1:40 dilution was incubated for 1 h at room temperature concealed from light. Alternatively the myofibrils were incubated with a Tmod-4 antibody (Proteintech, Chicago, IL) overnight at 4°C at a dilution of 1:200. After a rinse in RB, an anti-rabbit Texas Red (Sigma Aldrich, St. Louis, MO) was incubated for 1 h at room temperature concealed from light. Following the secondary antibody step, the coverslips were mounted on microscope slides containing one drop of glycerol mounting medium (Ringkob et al., 2004) and p-phenylenediamine to prevent the fluorescent signal from fading, and sealed with nail polish. Phase-contrast, phalloidin, and nebulin staining was performed on the same sarcomere. Tmod staining was performed separately on different myofibrils, because staining of all proteins was not possible using our microscope and filter combinations. When the slides had dried, they were imaged on a Nikon inverted microscope (Model Diaphot) with a 100× objective (phase contrast and fluorescence) or 40× (phase contrast, rest sarcomere length).

A cooled charge-coupled device (CCD) was controlled by IPLab V3.6 (Signal Analytics Corp., Vienna, VA). Ten myofibrils were selected for each animal and each muscle, with each myofibril containing at least six sarcomeres. TFL was defined as half of the distance between the phalloidin band in adjacent sarcomeres, while the nebulin and Tmod lengths were determined in the same manner. In the case of RSL, 10 myofibrils were selected, and the sarcomere length (SL) determined from a 10 sarcomere segment. To measure the actin thin filament, nebulin, and Tmod distances, images were rotated to a horizontal direction and a straight line was drawn from the center of the stain intensity at the free thin filament end to the same position in theadjacent sarcomere. RSL was measured in a similar manner, except that RSL was measured by drawing a straight line from Z line to Z line. Pixel length was converted to micrometers (one pixel was 0.05 microns at 100× magnification). Further procedural details can be found in Ringkob et al. (2004).

### Data analysis

Myofibril data was analyzed using the statistical package in Microsoft Excel, and a student's *t*-test was performed to determine significance. Significant difference was established at *p* < 0.05.

## Results

**Figure 2:**
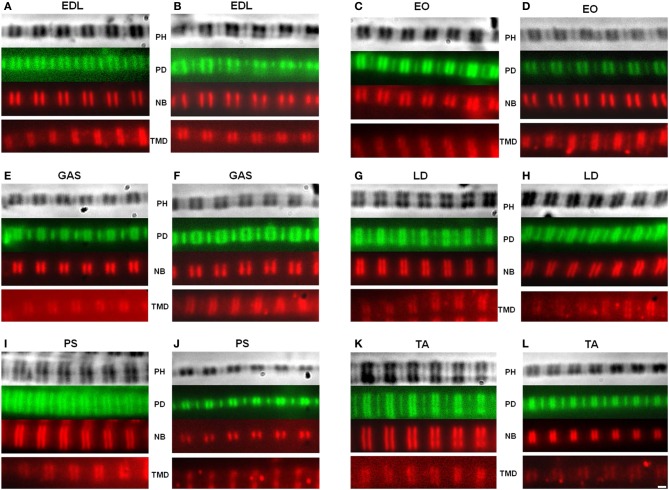
**Representative images of myofibrils**. The actin filaments were observed using fluorescein isothiocyanate (FITC), while the nebulin and tropomodulin doublets were measured using a Texas Red secondary antibody. A matched set of phase contrast, actin, and nebulin images were obtained. The tropomodulin image was from a separate myofibril with a similar sarcomere length. Top: phase contrast (PH) image of sarcomere; Second row: phalloidin (PD); Third row: nebulin (NB) antibody; Fourth row: tropomodulin-4 (TMD) antibody. Wild type **(A,C,E,G,I,K)**; homozygous mutant **(B,D,F,H,J,L)**. (EDL) Extensor digitorum longus, (EO) External oblique, (GAS) Gastrocnemius, (LD) Longissimis dorsi, (PS) Psoas major, (TA) Tibialis anterior. Magnification is 2000×.

Apparent titin sizes vary between different skeletal muscles. A representative agarose gel image showing the titin and nebulin region from several muscles (both WT and HM) is depicted in Figure 1B. As is evident, the HM rats showed an increased titin size across all muscles. It was previously reported that this mutation alters the normal titin splicing pattern in the heart, and the mutant titin molecules do not decrease in size with age (Greaser et al., 2008). Myofibrils prepared from muscles typically show a variety of SLs. Only those myofibrils that attained a sufficient SL to show doublet bands were used in data analysis to ensure that overlap of the thin filament free tips did not occur. In the FITC-phalloidin protocol, myofibrils were stained with or without a 4% formaldehyde fixation step. Without formaldehyde the fluorescent pattern shows a doublet at the edge of the H-zone (pointed end), as well as another band (barbed end) at the Z-disk (see Figure 2, second row). Conversely, formaldehyde fixation yielded a uniform phalloidin I-band staining with increased signal at the Z line. The addition or lack of a fixation step did not alter the phalloidin estimated TFL. A fluorescent signal with the NebN antibody was attainable after an hour of staining (Figure 2, third row), while the Tmod4 antibody required an overnight incubation step to obtain an adequate signal (Figure 2, bottom row). When analyzing the RSL, the striation patterns were readily apparent. For RSL data collection, the storage of muscle strips on the cable ties preserved their structure and allowed for easy manipulation.

Nebulin and Tmod antibodies were employed to determine the impact of titin splicing patterns on the thin filament system in skeletal muscle. It should be noted that the HM rats' skeletal titin size is identical across muscles (Li and Greaser, unpublished data). While these mutant titins are indeed significantly larger, they still express the N2A isoform observed in normal rats. All skeletal muscles in the adult mutant phenotype contained an equivalent-sized titin protein (~3.8 MDa) (Figure 1B). The wild type gel samples demonstrate a range in N2A isoform sizes (3.28–3.68 MDa).

**Figure 3:**
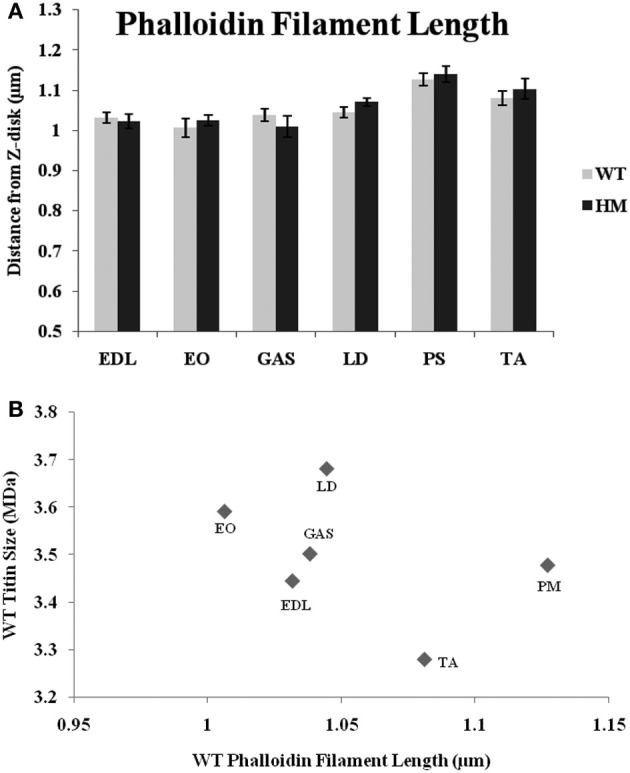
**(A)** Phalloidin staining determination of thin filament lengths in different muscles and rat genotypes. EDL, Extensor digitorum longus; EO, external oblique; GAS, gastrocnemius; LD, longissimus dorsi; PS, psoas major; TA, tibialis anterior; WT, wild type; HM, homozygous mutant. **(B)** Relationship between titin size and phalloidin estimated thin filament length.

For many years, phalloidin has been used to visualize actin in a variety of systems. Here, we use a FITC-labeled type to label the sarcomere's thin filament. In all muscles studied, there was no difference across genotypes in the distance from the Z-disk to actin's pointed end (Figure 3A). While there are length differences within a genotype, titin size had no apparent impact on TFL. Wild type rats' TFL ranged from 1.00 to 1.13 μm, and the HM rats' ranged from 1.01 to 1.14 μm, but there was no significant difference within each muscle. The shortest WT phalloidin length was found in EO, the shortest HM phalloidin length was GAS, but the PM, TA, and LD, respectively, showed the longest phalloidin length in both genotypes. The correlation between phalloidin length and adult WT titin size was very weak, indicating that titin size does not correlate with TFL (Figure 3).

**Figure 4:**
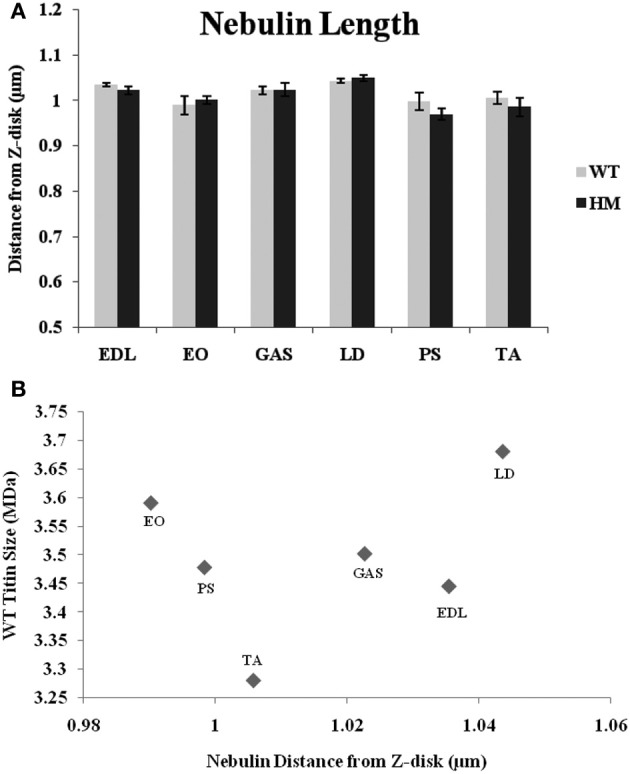
**(A)** Nebulin N terminus determination of tin filament lengths in different muscles and rat genotypes. See Figure 3 for muscle and genotype abbreviations. **(B)** Relationship between titin size and nebulin estimated thin filament length.

While nebulin, the second giant sarcomeric protein, contains numerous actin-binding sites along its length, it has remained unclear as to what role it plays in TFL regulation. A recent review outlines nebulin's potential models in regulating length (Littlefield and Fowler, 2008), but the complete mechanism remains unknown. To that end, immunofluorescence with a nebulin antibody (NebN) that targets the first three N-terminal modules (McElhinny et al., 2001) was performed. NebN stains in a doublet pattern close to, but not at, actin's barbed end (Castillo et al., 2009). The rat genotype did not affect nebulin's distance; there was no change across rat types. The agarose gel results also indicate that there is no change in nebulin size between genotypes (Figure 1B), and that the spontaneous mutation utilized here does not alter other sarcomeric proteins (Greaser et al., 2008). Therefore, it appears that altered titin size has no impact on nebulin size or the role nebulin plays in sarcomeric TFL determination. Unlike the values generated by phalloidin and the Tmod4 antibody, the nebulin distances varied much less across muscles (Figure 4A). The WT distances ranged from 0.99 to 1.04 μm, and the HM rats' ranged from 0.97 to 1.05 μm. The LD demonstrated the widest nebulin distance from the Z-disk in both genotypes. Each nebulin distance was shorter than that of the corresponding phalloidin distance, reinforcing nebulin's purported role as a stabilizing protein (Pappas et al., 2010). Further, when analyzing the relationship between nebulin distance and TFL, the correlation was low (*r*^2^ = 0.24, Figure 4B).

**Figure 5:**
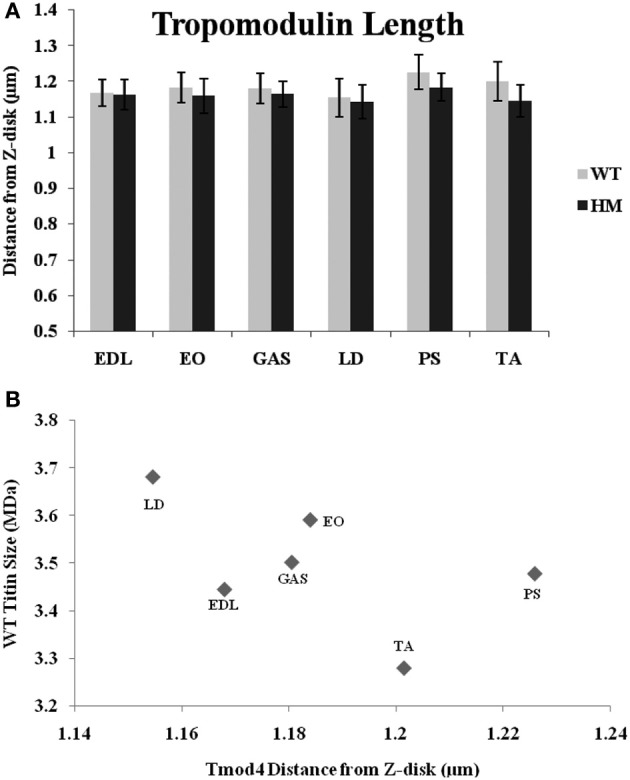
**(A)** Tropomodulin determination of thin filament lengths in different muscles and rat genotypes. See Figure 3 for muscle and genotype abbreviations. **(B)** Relationship between titin size and tropomodulin estimated thin filament length.

A Tmod-4 (the skeletal muscle-specific isoform) antibody was used to determine Tmod's distance from the Z-disk and to observe the relationship between Tmod, the actin filament, and titin size. If a stabilizing mechanism of nebulin is correct, Tmod may be responsible for setting a maximum TFL. The data shows that genotype did not affect Tmod distances, as there was no significant difference within muscle sets (Figure 5A). The wild type rat Tmod distances varied from 1.15 to 1.23 μm, while the HM rat's Tmod distances ranged from 1.14 to 1.18 μm. All Tmod distances were statistically similar, but PM showed the longest Tmod distance and LD demonstrated the shortest Tmod distance within their respective genotypes. Much like the nebulin distances, the Tmod data ranges are much smaller than those observed with phalloidin. This is in contrast to several other studies that showed wider ranges in Tmod distances (Fowler et al., 1993; Littlefield and Fowler, 2002; Castillo et al., 2009). There was a very weak correlation between Tmod and phalloidin distances (*r*^2^ = 0.13), but we did observe some common relationships in their lengths. In both the Tmod and TFL datasets, PM distances were the longest, but there was no relationship between other muscles' Tmod distances and their TFL. Every Tmod distance was longer than its corresponding phalloidin based TFL, which further supports the hypothesis that Tmod is responsible for specifying a maximum TFL. We also examined the relationship between Tmod distance and titin size, but found only a weak correlation (*r*^2^ = 0.29, Figure 5B). Excluding the PM, it appears that as the Tmod distance from the Z-disk decreases, the titin size increases. A previous study demonstrated a strong correlation between titin size and Tmod distance in rabbit and chicken skeletal muscle (Castillo et al., 2009). Additionally, the interaction between nebulin and Tmod (McElhinny et al., 2001) would suggest that they act in concert for TFL regulation. However, only a weak correlation (*r*^2^ = 0.17, across both genotypes) was found comparing nebulin and Tmod distances. It appears that the relationship between Tmod and nebulin is inversely related, such that there is more free F-actin as Tmod gets further from the Z-disk. The previous study showed a wider range of Tmod distances against nebulin and thin filament distances, but our results are indicative of a model that titin does not impact the Tmod-capping mechanism of the thin filament.

**Figure 6:**
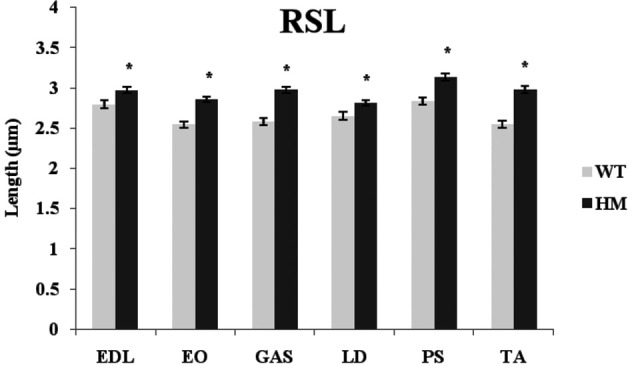
**Rest sarcomere length (RSL) in different muscles and rat genotypes**. See Figure 3 for muscle and genotype abbreviations. ^*^ *P* <0.05.

Titin performs a variety of functions in the sarcomere, including that of maintaining passive tension and a resting sarcomeric state. Presumably, titin size should be related to RSL and its physiological function. RSL was hypothesized to be a trait that was altered by this change in titin expression. The primary difference in the titin molecule between the WT and HM is found within the middle Ig and PEVK domains, its extensible regions. Because of the additional exons present in the mutant phenotype, the extensibility of the titin filament should increase and allow for longer SL. The longer titin protein in the mutant rat has shown a capability to reach longer SL without breaking (Greaser et al., 2008), and with this in mind we determined if the elongated titins would correlate with the longer RSL. The HM rats all showed significantly longer RSL compared to the WT, consistent with previous data from our lab. The addition of titin exons leads to elongated SL, and the HM rats demonstrated the effect that larger titin size has on SL (Figure 6). The WT lengths ranged from 2.55 to 2.84 μm, while the HM RSL ran from 2.81 to 3.13 μm. Because of this role titin performs, it was hypothesized that titin size should be related to RSL. However, there was no correlation between wild type RSL and adult rat titin size (*r*^2^ = 0.0002). The wild type PM had the longest RSL of its genotype (2.84 μm), despite containing one of the smaller titin sizes at 3.4 MDa, just as the HM PM had the longest RSL of its genotype (3.13 μm) (Figure 6). Wild type EO muscle has an intermediate titin size (3.5 MDa), but it had the shortest RSL. Conversely, wild type TA shows two isoform bands when resolved on an agarose gel, both of which are comparably smaller (3.2 MDa and 3.4 MDa). The wild type TA RSL is also at the small end of the RSL data range. The ranges of RSL in the HM were striking, since this genotype expresses titin isoforms of identical size. While LD muscle expresses one of the largest titin isoforms in the WT, the HM LD had the shortest RSL. The wide variety in titin sizes could explain the spread of RSL data, though the importance of the splice variants on RSL is not fully comprehended.

**Figure 7:**
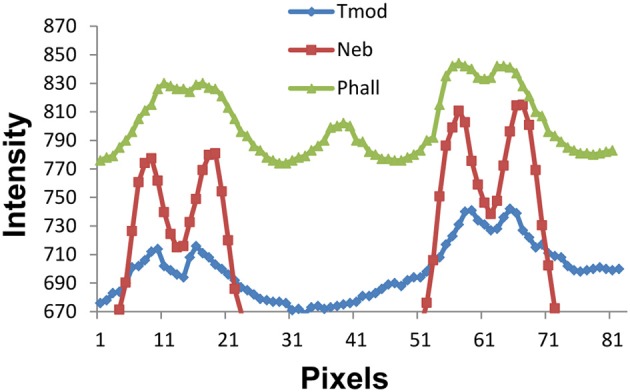
**Fluorescence staining intensity vs. pixel position plots for phalloidin, nebulin, and tropomodulin**. The Z line position is near pixel 40 (see phalloidin pattern for Z line peak) and the 2 minima between the nebulin, phalloidin, and tropomodulin doublets are at pixels ~13–15 and ~61–63, the centers of two adjacent sarcomeres. Note that the tropomodulin peak positions are 2–3 pixels farther from the Z line than those for nebulin, equivalent to 0.1–0.15 microns in length.

We are sensitive to the difficulty in measuring such small distances between the Tmod, nebulin, and actin thin filament locations. Since we are working near the edge of resolution of the light microscope, one may ask if there is sufficient resolution to distinguish 0.1 micron differences in filament length. A demonstration of the intensity vs. pixel position is shown in Figure 7. The phalloidin peak near pixel 40 is in the position of the Z line. The peak of the Tmod staining intensity is consistently 3–4 pixels farther from the Z line than either of the nebulin or phalloidin peaks. As long as there is no overlap of the thin filament tips, the width between filament staining positions do not change with SL.

## Discussion

Our data supports previous studies showing that Tmod appeared furthest from the Z-disk, and that the N-terminus of nebulin is shorter than that of the thin filament determined by Tmod staining (Castillo et al., 2009). Nebulin, therefore, should provide a minimum TFL and function as a stabilizer of the thin filament (Chen et al., 1993). The phalloidin and nebulin N terminus positions were very similar. Tmod localization is demonstrated past the end of the phalloidin-stained region, which could suggest that it is important in the dynamic nature of the actin filament (Littlefield et al., 2001; Li et al., 2009).

However, the crux of this study was to determine the effect of titin size on TFL. The relationship between these two factors could not be verified through immunofluorescence. There was no correlation observed, either comparing WT titin sizes to their respective TFL's, or comparing genotypes with drastically different titin sizes to their respective phalloidin lengths. Using the tibialis anterior muscle to demonstrate this point, the WT titin size is ~3.3 MDa and the Tmod length is 1.2 μm, while the mutant titin size is around 3.8 MDa and the Tmod length is 1.15 μm. The altered titin size does not seem to manifest a difference in TFL regulation and determination. Titin size and Tmod distance do not appear to be related. In rabbit skeletal muscles, Castillo et al. (2009) found a strong correlation between Tmod length and titin size. They hypothesize that TFL's are coordinated with titin size to provide an ideal contractile interconnection between the thick and thin filaments. We were curious, then, about what would happen to TFLs should titin size be subjected to manipulation.

The mutant rat model employed in this study provided us with the opportunity to investigate the effects of an enlarged titin phenotype upon its thin filament neighbors. This model has been the subject of several articles (Greaser et al., 2005, 2008), and it has been shown that this mutation is not subject to normal developmental titin isoform shifting. The mutation ablates the normal splicing pattern of the titin molecule, primarily changing the middle Ig and PEVK exon expression. As this is the main extensible region of the protein, force and tension are changed depending on the isoform size. Therefore, the impact that titin isoform alterations have upon the sarcomere can be further elucidated using this rat model. Size differences in rabbit skeletal muscle titins have been observed (Prado et al., 2005), as well as those in rat skeletal muscle (Ono, 2010). Titin interacts with a number of sarcomeric proteins (Linke, 2008), so a change in titin might herald structural changes in the accompanying myofibrillar proteins. As seen in the data presented above, however, this appears not to be the case.

We observed no change in apparent phalloidin lengths across genotypes (Figure 3A). Titin interacts with actin near its N-terminus, but not in the I-band region of titin (Trombitas et al., 1997), which may affect TFL. As there was no correlation between TFL and titin size, it would seem that they are assembled independently during myofibrillogenesis. Indeed, actin and non-muscle myosin are inserted prior to titin incorporation (Sanger et al., 2010); therefore, it stands to reason that titin and the thin filament do not impact each other. Of further interest is the fact that the PM showed the longest TFL in both genotypes, despite the large size difference between genotypes, further strengthening our hypothesis that titin size does not impact TFL in the rat. The titin size difference between genotypes does not dictate the TFL following fluorescent phallodin staining. As both phenotypes also demonstrated equal TFL variabilities, titin does not seem to be responsible for TFL determination.

Similar to titin, nebulin is a large, scaffolding protein that undergoes alternative splicing. However, we did not observe any changes in nebulin size across genotypes in the rat (Figure 1B). Nebulin and titin have been shown to interact with each via titin's PEVK domain (Ma and Wang, 2002), a hotspot for titin splicing. This may have lent credence to a scenario in which altered titin splicing impacts the titin-nebulin interaction; here, the mutation does not impact nebulin size or its N-terminus distance from the Z-disk (Figure 4A). Due to nebulin's large size and potential for splice variants, we found it curious that the mutation did not change the nebulin phenotype. Since all nebulin lengths were shorter than their respective TFL's, a model in which nebulin specifies minimum TFL seems feasible. Manipulation of nebulin expression levels have shown that down regulation causes shortened thin filaments (Bang et al., 2006; Witt et al., 2006; Gokhin et al., 2009; Pappas et al., 2010). The nebulin data ranges were also much smaller than the other studied proteins' distances, suggesting nebulin specifies a predetermined minimum TFL, regardless of the muscle. Ultimately, it does not seems that nebulin defines a maximum TFL, but that there may be another mechanism responsible for this to occur. There also seems to be no relationship between TFL and nebulin length, as there is no correlation between the two datasets. For example, the LD showed the furthest nebulin N-terminal distance from the Z-disk, even though it is in the mid-range of TFL's. As the LD contains a larger titin size in the WT animal, one could surmise that this is the reason why the nebulin distance is furthest; there was no correlation demonstrated between WT titin size and nebulin distance, though. It appears that the titin-nebulin interaction does not play a role in actin filament determination.

With nebulin providing a minimum length for the thin filament, Tmod performs a capping function on the actin filament's pointed end to prevent elongation (Weber et al., 1994). Titin has been implicated in determining TFL in the rabbit through a correlation between its size and with Tmod position (Castillo et al., 2009). We did not observe any justification for this claim, as there was no relationship between wild type rat titin size and Tmod distance from the Z line. Interestingly, the PM showed the greatest Tmod distance in both genotypes (Figure 5A), just as in the phalloidin length data above. Because the PM expresses one of the smallest titins in WT rat skeletal muscle, this does not support a hypothesized system in which Tmod and titin are related in establishing a specified TFL. Indeed, the data presented here show a weak negative correlation; that is, a shorter Tmod distance can be coupled to a larger titin size. There was also a weak negative correlation between Tmod distance and phalloidin length. It is possible that a closer relationship between titin size and thin filament may exist in other species, but we did not observe a good correlation in the rat. Ultimately, it does not seem that titin plays a major role in the thin filament structure of the myofibril.

As reported previously (Greaser et al., 2008), our mutant rat phenotype possesses a giant titin isoform (3.8 MDa) that persists through the animal's lifespan. Previous work showed that the RSL of cardiomyocytes was greater in mutants than WT (2.02 vs. 1.88 μm). Each RSL in the mutant phenotype was significantly larger (*p* < 0.05) than its WT counterpart, consistent with our previous report using cardiac muscle (Greaser et al., 2008). Significant differences were found within the mutant muscle set, which is surprising considering that all mutant skeletal muscles express the same titin size (Figure 1B). So even though the mutant titin sizes were identical and the WT sizes varied from 3.28 to 3.68 μm, the ranges in RSL were similar, at 0.29 μm for the WT and 0.32 μm for the mutant. The increase observed in the homozygote mutant phenotype is most likely the result of the decreased splicing manifested primarily in the extensible I-band region of titin. Because the middle Ig and PEVK regions are more expansive in our rat model, this facilitates an increase in RSL. There was no correlation between WT titin size and RSL, though. Regardless, the data presented here demonstrate the effect that titin confers upon SL. With the addition of extensible domains in titin's band region, increased SLs are possible. More research is needed to fully comprehend the implications of this giant titin isoform in the striated muscle system.

Actin TFL has been demonstrated in numerous studies to be influenced by two sarcomeric proteins: the giant protein nebulin and the pointed-end capping protein Tmod. Our data indicates that titin does not alter TFL, but supports a system in which TFL is influenced by nebulin and Tmod. The current hypothesis is that nebulin controls a minimum TFL, while Tmod may be responsible for defining a maximum TFL. Despite earlier studies suggesting an interaction between nebulin and Tmod (McElhinny et al., 2001), more recent evidence shows this binding to be relatively weak (Weber et al., 1994). Because the distance between the nebulin signal and the Tmod signal is as high as 0.23 μm in rat skeletal muscle, it seems unlikely that these two proteins interact directly to influence TFL. Therefore, it appears that nebulin and Tmod act independently to control TFL.

The rat mutation model employed in this study impacts the splicing state of titin. While titin is able to bind to actin (Trombitas et al., 1997; Kulke et al., 2001; Nagy et al., 2004; Raynaud et al., 2004; Chitose et al., 2010) and nebulin (Ma and Wang, 2002; Witt et al., 2006), the alternatively-spliced titin isoforms do not modify the thin filament configuration. Additionally, no interactions between titin and Tmod have been noted to date, which supports the hypothesis that nebulin and Tmod are the chief agents in TFL determination. The data presented in this study show that titin does not affect TFL but is related to RSL. A 3.8 MDa titin isoform alters some features of the sarcomeric machinery, but this giant protein does not appear to control the actin TFL.

We are still left with the dilemma of explaining the reason for the difference in conclusions between the Castillo et al. (2009) and the current study. A careful re-examination of the molecular weights used for the correlation from the Prado et al. paper (Prado et al., 2005) with those determined in our own laboratory revealed some significant differences. Castillo used the following numbers: Psoas—3295 mDa; TA—3414; EDL—3425; Gast—3435; Sol—3596; PD—3655; Dia—3700. Our own values for some of these muscles, determined by agarose SDS electrophoresis (Warren et al., 2003), were significantly different: Psoas—3300 MDa; TA—3300; EDL—3570; Gast—3460; Sol—3588; PD—3557; Dia—3675. Using the TFL from Castillo and constructing a new correlation, the number decreased to 0.52 with only 25% of the variation being explained. Thus we feel that the previous titin- thin filament length relationship may have been fortuitous and partially based on incorrect molecular weights. The question of the titin relationship may need to be resolved with a wider group of muscles.

### Conflict of interest statement

The authors declare that the research was conducted in the absence of any commercial or financial relationships that could be construed as a potential conflict of interest.
